# Detection of Breast Cancer Lump and BRCA1/2 Genetic Mutation under Deep Learning

**DOI:** 10.1155/2022/9591781

**Published:** 2022-09-19

**Authors:** Yue Miao, Siyuan Tang

**Affiliations:** Department of Computer Science and Technology, Baotou Medical College, Inner Mongolia University of Science and Technology, Baotou 014160, China

## Abstract

To diagnose and cure breast cancer early, thus reducing the mortality of patients with breast cancer, a method was provided to judge threshold of image segmentation by wavelet transform (WT). It was used to obtain information about the general area of breast lumps by making a rough segmentation of the suspected area of the lump on mammogram. The boundary signal of the lump was obtained by region growth calculation or contour model of local activity. Meanwhile, multiplex polymerase chain reaction (mPCR) and mPCR-next-generation sequencing (mPCR-NGS) were used to detect BRCA1/2 genome. Sanger test was used for newly high virulent mutations to verify the correctness of mutagenic sites. The results were compared with the information marked by experts in the database. According to Daubechies wavelet coefficients, the average measurement accuracy was 92.9% and the average false positive rate of each image was 86%. According to mPCR-NGS, there was no pathogenic mutation in the 7 patients with high-risk BRCA1/2 genetic mutations. Single nucleotide polymorphism (SNP) in nonsynonymous coding region was detected, which was consistent with the Sanger test results. This method effectively isolated the lump area of human mammogram, and mPCR-NGS had high specificity and sensitivity in detecting BRCA1/2 genetic mutation sites. Compared with traditional Sanger test and target sequence capture test, it also had such advantages as easy operation, short duration, and low cost of consumables, which was worthy of further promotion and adoption.

## 1. Introduction

The incidence of breast cancer has been on the rise since the second half of the 20^th^ century, and it has become a major health problem for women [1]. That the breast lesions are detected, discovered, and diagnosed as early as possible is the most effective way to save the patient's life [2]. Mammography is one of the most important methods for early detection of breast cancer worldwide [3]. With the advance of time and the rapid development of modern science and technology, the computer-assisted testing technology of breast cancer has become a hot subject that attracts more scholars' attention [4]. Computer-assisted examination technology can not only save much human labor, but also help to understand and analyze mammogram images [5]. The causes of breast cancer are complex. Generally speaking, it is mainly because the cells and tissues in the breast change their normal characteristics under the combined influence of internal and external carcinogenic factors, thus forming abnormal cell proliferation phenomenon. When these lesions reach their maximum recovery rate, breast cancer occurs [6–8]. Breast cancer is the most deadly gynecological malignant tumor at present. According to the document research, BRCA1/2 genetic mutation exists in more than 80% of patients with high genetic breast cancer, and the incidences of lifetime breast cancer and breast cancer with this genetic mutation are much higher than the general population [9, 10]. Hence, it is necessary to monitor BRCA1/2 genetic mutation and give the corresponding intervention in high-risk groups.

At present, the screening and early treatment of breast cancer mostly rely on modern techniques such as mammography, magnetic resonance imaging (MRI), and ultrasonic examination [11]. With the advantages of simple operation, high cost performance, and noninvasive surgery, mammography has become the preferred photographic method in breast cancer screening [12]. With the rapid development of medical technology and modern scientific and technological means, the early treatment of breast cancer also plays an increasingly great role in women's health care [13]. In the design of computer-aided diagnosis (CAD) system, how to reasonably classify the range of lesions is a key skill [14]. Since the middle of the twentieth century, the concept of image segmentation has emerged in the field of computer image processing. In the following decades, image segmentation has become a hot topic in the field of digital image processing [15, 16]. Image subdivision method is classifying the image into a considerable number of regions with unique properties and extracting the interest region of the technical method and process [15]. In the field of biomedical application, image segmentation technology plays a crucial role in medical image. It can help to measure and locate the tissue structure of cancer and other diseases and make it easier to measure the tissue volume [17].

To sum up, at present, how to diagnose breast cancer in initial stage is a major problem that needs to be paid attention to in clinic. An innovative separation method based on wavelet transform (WT) was proposed. The algorithm effectively classified the suspicious area of the breast image. Then, according to the characteristic parameters of the tumor area, it also found the tumor tissue in the breast accurately, thereby realizing the automatic detection of the breast tumor. Meanwhile, multiplex polymerase chain reaction-next-generation sequencing (mPCR-NGS) was performed on eligible high-risk group with BRCA1/2 genome mutation to test whether there was BRCA1/2 genome mutation, and satisfactory conclusions were obtained.

## 2. Materials and Methods

### 2.1. Objects of Study

Seven patients who were the suspected high-risk BRCA1/2 genetic mutation carriers of XX Hospital from June to December 2020 were included. Among them, there were 4 patients who were diagnosed with breast cancer and 3 patients who were asymptomatic high-risk carriers. All the patients signed the informed consent, and the research was approved by the ethics committee.

The inclusion criteria were as follows: I. patients whose immediate family members had breast cancer lesions; II. patients whose family member had breast cancer; and III. patients whose onset age of early-onset breast cancer was less than or equal to 36 years old. If one of the above three conditions was satisfied, the research was implemented. The exclusion criteria were as follows: I. patients who refused to sign or did not sign the informed consent; and II. patients who had the medical history of trauma, cardiovascular disease, and metabolic disease.

### 2.2. The Serial Number of Double-Blind Method

The peripheral blood samples of the research objects and the control group were coded as *P*1, *P*2, *P*3, *P*4, *P*5, *P*6, *P*7, and *P*8. After the sequencing, the third party finished the unblinding task.

### 2.3. Extraction of Deoxyribonucleic Acid (DNA) of Peripheral Blood

At the temperature of 200°C to 250°C, the samples were extracted out and placed on the centrifugal tube plate rack, and they were thawed at room temperature. 20 *μ*L proteinase K and 200 *μ*L anticoagulant peripheral venous blood solution were added to 1.6-mL centrifuge tubes, and they were gently flicked for several times. After the complete uniformity, instantaneous centrifugation was performed. After the covers of the tubes were removed, 4 *μ*L ribonuclease A (RNase A) was added to each tube and the total vorticity was 15 s. After instantaneous centrifugation, the tubes were left standing for about 2 min at room temperature. 200 *μ*L Buffer AL was added and the vorticity was 15 s. After incubation at 56°C for 10 min, the samples were removed from the metal bath and cooled to normal temperature for 2 min. 200 *μ*L anhydrous ethyl alcohol was added and mixed with vorticity oscillation for 15 s. Then, the centrifugation was performed instantaneously. After centrifugation, the solvent was transferred to the purification column, avoiding the contact with the nozzle of the purification column as far as possible. After the centrifugation at 6,000 rpm for 1 min, the purification column was transferred into a new collection catheter, and another 500 *μ*L Buffer AW1 was added to the purification column for elution. After the centrifugation at 6,000 rpm for 1 min, the waste liquid was discarded, and the purification column was transferred into a new collection catheter. 500 *μ*L Buffer AW2 was added to the purification column for elution. After the centrifugation at 20,000 rpm for 3 min, the waste liquid was discarded. Then, the residual liquid at the end of the tube was wiped with absorbent paper, and it was centrifuged at 20,000 rpm for 1 min. A new 5-mL centrifuge tube was taken, into which the purification column was transferred. 200 *μ*L Buffer AE was added. Subsequently, after the cover of the centrifuge tube was closed, it was placed at the indoor humidity for 2 min, and the centrifugation was performed at 6,000 rpm for 1 min. Finally, the liquid in the centrifuge tube was collected, which was the obtained gDNA.

### 2.4. The Technology of mPCR to Amplify BRCA1/2 Gene

5 *μ*L PCR amplification product was taken and mixed with 1 *μ*L 6 × Loading buffer. The sample was added to 0.6% agarose gel pore by pipetting gun. The amount of foam was minimized and the liquid was made to overflow the pore. Additionally, 3 *μ*L 1 K DNA Marker was added into the pore for the molecular number marker. According to the direction of the positive and negative electrodes, the groove cover was closed, and the power switch was turned on. The voltage was adjusted to 120 V for constant pressure electrophoresis, and the duration of agarose gel electrophoresis was set to about 30 min. After the electrophoresis was completed, the power switch was turned off immediately. The gel was carefully extracted from the electrophoresis tank, and it was put into the two-dimensional gel electrophoresis image analyzer for image scanning observation and inspection. Bright and clear amplification bands were obtained by 1 K DNA Marker, which were labeled with the size of 1 Kb–10 Kb. The electrophoresis bands of PCR products were observed to check the location and brightness of target bands and compared with Marker. If the bands of the amplification products were concentrated in the region of 2 K–7 K, the amplification was successful.

### 2.5. Construction of the Resequencing Library for the Second-Generation Sequencing

5 *μ*L amplified material and 1 *μ*L 6 × Loading Buffer were evenly stirred and added to the wells of 2% agarose gel, with 3 *μ*L 100 bp DNA Marker as the molecular weight marker. Constant pressure electrophoresis was performed at 120 V for nearly 25 minutes. The size of the electrophoretic band of each PCR product was carefully observed. If the interruption band was found in the size of 202 bp–423 bp, the interruption was successful.

### 2.6. Designing Primers for Mutation Sites

According to the mutation sites obtained by mPCR-NGS sequencing, the first-generation sequencing experiments were used for nonsynonymous single nucleotide polymorphism (SNP) (serial numbers were 1, 2, and 3) with low mutation frequency. Primers were designed by the primer premier 5.0, and the product width of primer amplification generally ranged from 400 bp to 750 bp.

### 2.7. Segmentation of Lump Based on WT

Any signal is described as a sum of string functions according to Fourier theory. Hence, when a signal is completely described by the Fourier function, it has a spectral resolution rather than a temporal resolution, which means that the frequencies involved in the message can be identified, but the time at which it occurs cannot be identified. In the 1980s, some French physicists invented the wavelet analysis theory to inherit the advantages of the traditional Fourier analysis method and overcome the deficiencies [18, 19]. Through the development of recent decades, the foundation of modern wavelet analysis theory has been basically laid. Moreover, wavelet analysis has not only become a new field of applied mathematics, but also started to be used in many engineering fields.

The WT coefficient refers to a specific waveform, which is unique in that the wavelets have a finite width and an average value of 0. It borrows from Fourier analysis to decompose an electronic signal into a series of overlapping sinusoids of different frequencies. Wavelet analysis resolves the signal into a series of overlapping wavelet functions, which are derived from a set of identical mother wavelet functions through corresponding transformations. Wavelet analysis helps to obtain the features of the date signal when the signal occurs, which is usually achieved by using the translation of mother wavelet. Similarly, the frequency features of the time signal are obtained by scaling the WT.

WT is classified into the continuous WT and the discrete WT. For continuous WT, all *y(s)* in L^2^(*R*) space are extended under the wavelet basis, which is called *y(s)* continuous WT. In equation (1), *m* represents zoom factor and *γ* represents time translation.

(1) $$\begin{array}{c} U_{\left(m,\gamma \right)}=<y\left(s\right),\theta_{m,\gamma }\left(s\right)\geq \frac{1}{\sqrt{m}}\int^{}Ry\left(s\right)\bar{\theta \left(s-\frac{\gamma }{m}\right)}d{}_{s}. \end{array}$$

For the discrete WT, in any *L*^2^(*R*) space, the discrete WT of *y(s)* is expressed by the (2) and (3). It is important to note that this discrete transformation is about continuous large-scale parameters and translational parameters, not about *s*.

(2) $$\begin{array}{c} U_{Z}\left(e，i\right)={\int^{}}_{Y}y\left(s\right)\bar{{∁}_{hl}\left(s\right)}ds, \end{array}$$

(3) $$\begin{array}{c} {∁}_{hl}\left(s\right)=\frac{1}{\sqrt{2^{h}}}∁\left(\frac{s}{2^{h}}-i\right). \end{array}$$

WT is based on Fourier transform, which is further developed. It inherits the advantages of Fourier transform and overcomes the shortcomings. Generally speaking, WT has both frequency parsing and time parsing. Compared with Fourier transform, one of the biggest advantages is judging the timing of specific phenomena in the signal. Furthermore, it is possible to extract different resolution features from information because of the multiscale features of WT. Finally, in terms of the operating rate of the WT, it is much faster than Fourier transform.

### 2.8. Automatic Segmentation of Lump Based on Gray Histogram WT

The gray histogram of mammogram was classified by WT, and the segmentation threshold was determined regarding the limit value of WT modulus. First, before the lump area was extracted, the influence of noncancerous parts of the breast on the determination of the lump area was eliminated, including the capillaries in the breast and the scars on the skin. The wavelet filtering method was selected as the main method to achieve image smoothness. Consequently, the effect of texture signals such as capillaries and skin in breast images on the tumor detection results was reduced. After the smoothing filtering, the histogram of the breast image needed to be recalculated. For most medical images, before the histogram transformation was completed, the image was normalized and the gray scale range was controlled into the gray space of 0–225, because the gray level area was large and the gray histogram distribution was inconsistent. In (4), *P* represented the prenormative image, and *K* represented the image processed by the specification.

(4) $$\begin{array}{c} K\left(z,u\right)=\left(P\right.\left(z,u\right)-\text{min}\left(P\right)\times \frac{255-0}{\text{max}\left(P\right)-\text{min}\left(P\right)}. \end{array}$$

In the breast image, there was usually a jumping in gray value where the lump area was connected to the nearby normal tissue area. Hence, only by finding the gray value of the distortion correction point was the whole breast separated. However, in the actual breast images, since the volume of the lump department was relatively small compared with the whole breast department, there were some small bumps in the high point of the gray order in the gray histogram, which were not observed directly. The wavelet for singularity test was obtained from smooth function based on the principle of wavelet transform modulus maximum test. If *δ(z)* was the smooth function with low pass features, the first stage of derivative was expressed as

(5) $$\begin{array}{c} \omega \left(z\right)=\frac{d\delta \left(z\right)}{ds}. \end{array}$$

It was used as a wavelet, and the (6) was obtained.

(6) $$\begin{array}{c} Q_{d}^{1}\left(a,z\right)=f\left(z\right)*\theta_{a}^{1}\left(z\right)=s\frac{d}{dz}\left[f\left(z\right)*\varphi_{a}\left(z\right)\right]. \end{array}$$

(7) represents the scaling transformation of *δ(z)* under scale *S*.

(7) $$\begin{array}{c} \varphi_{a}\left(z\right)=\frac{1}{a}\varphi \left(\frac{z}{a}\right). \end{array}$$

In (6), the WT of the signal *f(z)* was expressed as a derivative of *f(z)* after smooth processing at the scale *S*. At this point, the extreme point of the WT was the turning point of *f*(*z*)*∗δ*(*z*), which was the step point in the extreme case. *Q*_*d*_^1^(*a*, *z*) was used to represent the sign signal time. The maximum point corresponded to the place where the information changed dramatically, while the minimum point corresponded to the place where the information changed slowly. This special property of WT was used to locate the singular points in the gray histogram of breast image to find the corresponding singular points with small gray order value. The minimum point that was nearest to the singular point was found, and the corresponding gray scale threshold point of the singular point was the threshold point of lump suspected region division after scale regression. The actual size and morphology of the lump were considered, and the addition of the area information of the block and the ratio of length to width of the minimum enclosing rectangle became another restriction requirement for image segmentation. According to the above requirements, the general situation of the suspected range of the lump was obtained, and the range of the lump in the mammography was generally detected.

The gray histogram of mammogram was examined by using wavelet mode maximum method. After the segmentation threshold was found and the lump location was located, the lump location was observed to have different properties. Some lumps stuck to the surrounding tissues, so it was difficult to precisely locate the boundaries of these lumps. There were some obvious differences between the other parts of the tumor and the surrounding tissue boundaries. For those lumps with obvious differences in gray features between the lump area and the surrounding tissue, simple region growth method was used to obtain the boundaries of these lumps. For those lumps with fuzzy edges, active contour model was used to extract their boundaries. (8) was the contour model equation of local activity.

(8) $$\begin{array}{c} G\left(B,j_{1},j_{2}\right)=\omega R\left(B\right)+wT\left(B\right)+\sigma_{1}\underset{\text{inside}\left(B\right)}{\int }{\left|H-j_{1}\right|}^{2}epeq+\sigma_{2}\;\underset{\text{inside}\left(B\right)}{\int }{\left|H-j_{2}\right|}^{2}epeq. \end{array}$$

The parameters used in the contour model of local activity changed with the change of each point, and it was not a long-term invariant gray scale mean value of the target region or background region. Therefore, when there was a certain regional difference between the homogeneity of the target area and the background area, the image was well separated through this mode. Hence, the contour mode of local activity helped to solve the deficiency of image separation in CV mode where the homogeneity of two edges was different. With the introduction of local information, the boundary of the target region was accurately found when the problem of image separation with uneven gray scale distribution was solved.

## 3. Results

### 3.1. Sequencing Quality and Coverage Quality

Table 1 shows the quality detection of the sequencing samples.

**Figure 1:**
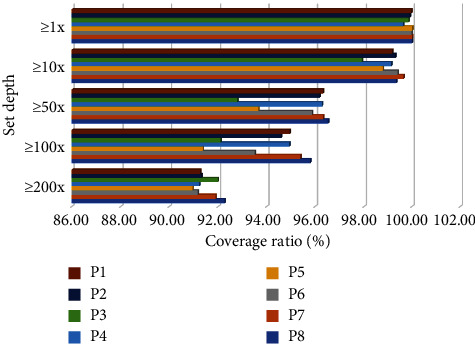
The tests of the coverage depth of experimental group and control group.

Figure 1 shows the tests of the coverage depth of the samples.

### 3.2. Analysis of Sequencing Results

Table 2 shows the results of sample sequencing.

According to the detection results, no pathogenicity mutation sites with a low occurrence rate (<1%) in Asian population were detected.

### 3.3. The Verification Results of the First-Generation Sequencing

The recent generation of gene detection was confirmed by the nonsynonymous SNP with low mutation frequency in three Asians and high occurrence frequency in the samples, which was consistent with the results of sequencing (Table 3).

**Figure 2:**
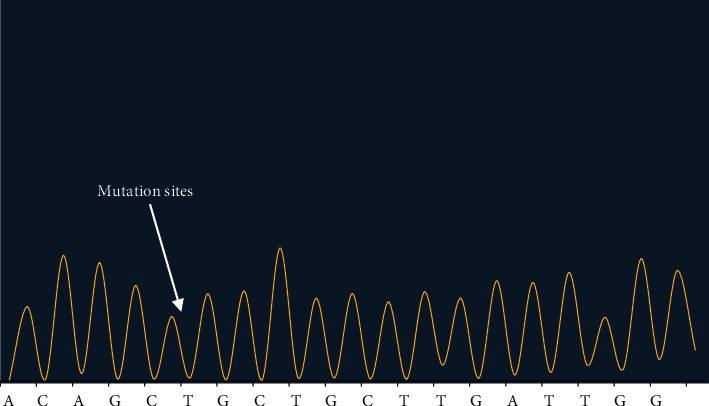
The first-generation sequencing was used to test sample *P*2 at site NO. 1, and the location of mutation sites was c.7397T>G. BRCA2 gene belonged to the positive chain, so the mutation of this site was A>G according to the principle of reverse complementation. The homozygosis was that one hundred percent of the mutation occurred.

**Figure 3:**
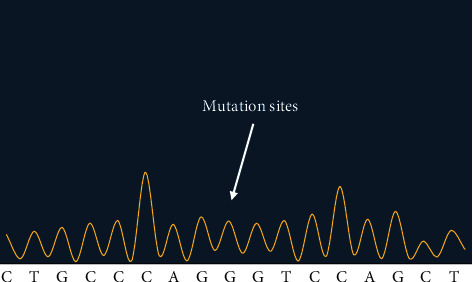
The first-generation sequencing was used to test sample *P*5 at site No. 2, and the location of mutation sites was c.4900A>G. BRCA1 gene was in negative chain, so the mutated gene was at this position A>G. The homozygosis was that one hundred percent of the mutation occurred.

**Figure 4:**
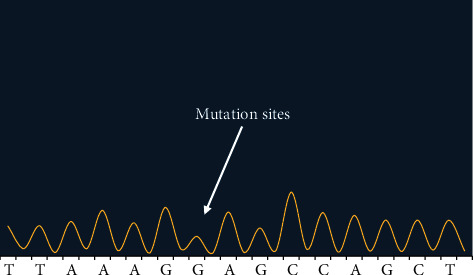
The first-generation sequencing was used to test sample *P*6 at site NO. 3, and the location of mutation sites was c.3113A>G. BRCA1 gene was in negative chain, so this site was A>G after mutation. It was the heterozygosis that the target gene mutated into bimodal type.

Figures 2–4 showed the sequencing results of specific mutation sites.

### 3.4. Results of Automatic Segmentation of Lumps by WT

**Figure 5:**
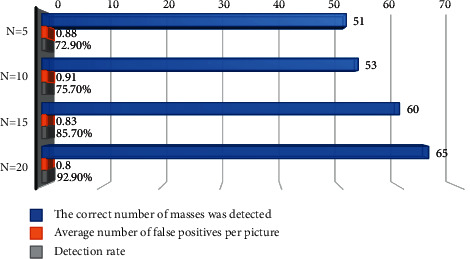
Comparative experiments of four WTs with different sizes.

To obtain the best experimental efficiency, four WTs with different sizes were selected for comparative experiments. After the test results were analyzed, the wavelet parameters to obtain the best segmentation effect were determined.  Figure 5 shows the comparative test results.

### 3.5. Results of Lump Edge Detection Based on Active Contour Model

Among the 70 breast images that included 68 lumps, 66 lump areas were obtained by rough examination, and 59 of them were completely identified and their boundaries were extracted.

## 4. Discussion

The mPCR-NGS was performed on seven clinically collected high-risk BRCA1/2 mutation carriers, and no pathogenicity mutation sites were found. Nevertheless, through the first-generation genetic sequencing, the experimental results were consistent with the results of sequencing proposed in the study. At the stage of rough segmentation of breast lump, the gray histogram of each image was firstly obtained, and the distribution of gray histogram of each image was also different with the difference of specific conditions in the imaging. Besides, it was necessary to implement the gray histogram standardized management.

The WT of Daubechies with the orders including *N* = 5, *N* = 10, *N* = 15, and *N* = 20 was selected to perform one-dimensional WT. The results showed that the WT of the order *N* = 20 obtained the best measurement effect, the detection rate was more than 92.90%, and the average number of false positive expression in each image was 0.86. According to the theoretical analysis, since the white noise was singular everywhere in the whole signal, it was pointed out that the amplitude and density of the maximum point of WT mode formed by white noise were inversely proportional to the large-scale parameters of wavelet. Nonetheless, the actual image information was different. The singularity of image information was mainly caused by the incoherence of image gray, and the maximum value of WT was little affected by the size change. With the influence of small-scale WT, it was difficult to distinguish the noise from the detail information of the signal because the dominant function of the maximum point formed in the detail signal of the image was similar. Moreover, it was why the detection accuracy was low and the false-positive rate was high when the small size was used. On the contrary, with the influence of the minimum wavelet function of large size, the mutation signal of the gray level of image had great effect on the maximum dominance of the whole signal. Therefore, the selection of large size wavelet parameters produced good singular value detection features, thus helping to achieve good efficacy in rough segmentation of lumps.

Additionally, although the selected WT with *N* = 20 still retained a relatively high false-positive rate, the possible reduction in the number of false-positive expressed lumps in subsequent tests was considered. The wavelet of *N* = 20 was chosen as the coefficient of WT and analyzed theoretically. The WT of order *N* = 20 had good processing efficiency for different wavebands, and it was helpful to cut the evenly distributed gray order histogram exactly. After the appropriate wavelet was selected, the odd features in the gray histogram were measured by using the basic principle of the modulus maximum of WT. Furthermore, this singularity corresponded to the gray distortion in the straight mammary gland image. After the appropriate singular loci was determined and the appropriate segmentation threshold was selected, the rough segmentation was performed automatically according to the segmentation threshold. In this experiment, 70 images from the MIAS database were examined. The results showed that the proposed method satisfactorily determined the size of hard lumps in mammogram, and the detection rate was 92.9%. As for undetectable lumps, there were generally two cases. The first case was that during the imaging process, the lump area and the pectoral muscle area were double merged and eliminated during the pretreatment process. The second case was that it was difficult to detect the lump in breast images because the gray data were very similar to those of the surrounding normal tissues. However, as for the lumps detected by rough segmentation, the proposed method was helpful to well extract the boundary of lumps.

To sum up, this method basically achieved the automatic segmentation of digital mammography lumps by computer, and the segmentation effect was relatively ideal, which helped to promote the value of further study and adoption. In the modern society with the continuous development of high technology, people pay much attention to the diseases that they suffer from, and they have a comprehensive understanding of how to prevent and treat diseases [20]. In the process of collecting samples, both patients with breast cancer and specialist doctors had a strong interest in genomic screening and an emerging technical field, in which they voluntarily participated. It fully demonstrated that there was further development space in the field of genetics [21].

## 5. Conclusion

The techniques of breast tumor segmentation in mammography were systematically summarized and analyzed. After the study of various references, a new method for mammogram lump segmentation was provided. The principle of wavelet maximum was used to measure the singular points of high gray histogram blocks in breast image, and the rough segmentation threshold of hard blocks was determined by selecting appropriate singular points. Moreover, the specificity and sensitivity of BRCA1/2 genetic mutation sites were all detected by mPCR-NGS sequencing platform for clinically high-risk BRCA1/2 mutation carriers. Compared with traditional Sanger sequence testing and target sequence testing methods, mPCR-NGS sequencing platform had such advantages as easy execution, short time consuming, and low cost.

Any early diagnosis and prevention of breast cancer has always been the focus of biomedical research. Nevertheless, due to the limitations of research equipment and technology, the tumor isolation and genetic mutation detection methods on mammography images were investigated preliminarily. There are still many advanced algorithms that are not used, and there is still room for improvement and optimization in the experimental results. Additionally, there is still much work to be done in the future.

## Figures and Tables

**Table 1:** Quality detection of samples.

Sample	*P*1	*P*2	*P*3	*P*4	*P*5	*P*6	*P*7	*P*8
Map bases	298237468	296811389	396791957	297232536	197714073	290648699	294785522	290241136
Clean bases	636832098	598907146	794252182	697338797	494135783	694778908	699269869	594654691
Clean reads	73558937	61226224	67547282	76173306	55717899	60804209	69899226	65224092

**Table 2:** Results of sample sequencing.

Sample name	*P*1		*P*2		*P*3	*P*4
Gene	BRCA2	BRCA2	BRCA1	BRCA1	BRCA1	BRCA2
Allele Freq Asn	23	120	36	36	36	100
Coordinate	39382638	30264927	419200372	43375502	41172649	32929387
HGVSc	c.H75A>C	c.6634T>C	c.4373A>G	c.2643A>G	c.237C>T	c.7397T>C
COSMIC ID	COSM103751		COSM134096		COSM166528	
PolyPhen	Benign (0.536)	Benign (0.231)	Benign (0.1)	Possibly damaging (0.732)	Benign (0.231)	Benign (0)
Geno type	horn	het	hom	het	hom	hom

Sample name	*P*5	*P*6	*P*7	*P*8	Control group	

Gene	BRCA2	BRCA1	BRCA2	BRCA1	BRCA1	BRCA2
Allele Freq Asn	100	33	100	26	100	100
Coordinate	33683462	43477289	32352679	32647899	30038853	31125674
HGVSc	c.72378>C	c.7532AXJ	c.2367T>C	c.7792A>C	c.2352T>C	c.1567T>C
COSMIC ID		COSM13783		COSM 14202		
PolyPhen	Benign (0.001)	Benign (0)	Benign (0.343)	Benign (0)	Benign (0.1)	Benign (0.116)
Geno type	hom	het	hom	het	hom	hom

**Table 3:** Site information of the first-generation sequencing.

Sample	*P*2	*P*5	*P*6
Gene	BRCA1	BRCA2	BRCA1
HGVSp	p.Serl 521Gly	p.Val2688AIa	p.Glul236Gly
HGVSc	c.1524A>G	c.8745T>C	c.1624A>G
Coordinate	42724678	39095326	42456093
	rs31566633	rs44291	rs773092

## Data Availability

The raw data supporting the conclusions of this article will be made available by the authors, without undue reservation.
